# High-Yield Production of Polyhydroxybutyrate and Poly(3-hydroxybutyrate-*co*-3-hydroxyhexanoate) from Crude Glycerol by a Newly Isolated *Burkholderia* Species Oh_219

**DOI:** 10.3390/polym17020197

**Published:** 2025-01-14

**Authors:** Suk-Jin Oh, Gaeun Lim, Yebin Han, Wooseong Kim, Hwang-Soo Joo, Yun-Gon Kim, Jae-Seok Kim, Shashi Kant Bhatia, Yung-Hun Yang

**Affiliations:** 1 Department of Biological Engineering, College of Engineering, Konkuk University, Seoul 05029, Republic of Korea; equal73@naver.com (S.-J.O.); lge0919@naver.com (G.L.); hanyebin3@gmail.com (Y.H.); shashikonkukuni@konkuk.ac.kr (S.K.B.); 2 College of Pharmacy and Graduate School of Pharmaceutical Sciences, Ewha Womans University, Seoul 03760, Republic of Korea; wooseong_kim@ewha.ac.kr; 3 Department of Biotechnology, College of Engineering, Duksung Women’s University, Seoul 01370, Republic of Korea; hwangsoojoo27@duksung.ac.kr; 4 Department of Chemical Engineering, Soongsil University, Seoul 06978, Republic of Korea; ygkim@ssu.ac.kr; 5 Department of Laboratory Medicine, Kangdong Sacred Heart Hospital, Hallym University College of Medicine, Seoul 05355, Republic of Korea; jaeseok@hallym.ac.kr; 6 Institute for Ubiquitous Information Technology and Applications, Konkuk University, Seoul 05029, Republic of Korea

**Keywords:** polyhydroxyalkanoates, waste glycerol, biodiesel, *Burkholderia*

## Abstract

Crude glycerol (CG), a major biodiesel production by-product, is the focus of ongoing research to convert it into polyhydroxyalkanoate (PHA). However, few bacterial strains are capable of efficiently achieving this conversion. Here, 10 PHA-producing strains were isolated from various media. Among them, *Burkholderia* sp. Oh_219 exhibited the highest polyhydroxybutyrate (PHB) production from glycerol and was therefore characterized further. *Burkholderia* sp. Oh_219 demonstrated significant tolerance to major growth inhibitors in CG and metabolized the fatty acids present as impurities in CG. Furthermore, the Oh_219 strain was genetically engineered using *phaC*_BP-M-CPF4_ and *phaJ*_Pa_ to enable the fatty acid-based production of poly(3-hydroxybutyrate-*co*-3-hydroxyhexanoate) (PHBHHx), a component of CG. The resulting strain produced PHBHHx containing 1.0–1.3 mol% of 3HHx from CG. Further supplementation with capric and lauric acids increased the 3HHx molar fraction to 9.7% and 18%, respectively. In a 5 L fermenter, the Oh_219 strain produced 15.3 g/L PHB from 29.6 g/L biomass using a two-stage fermentation system. This is the highest yield reported for PHA production from glycerol by *Burkholderia* spp. Additionally, PHB produced from CG had a lower melting point than that from pure glycerol and fructose. Taken together, *Burkholderia* sp. Oh_219 is a promising new candidate strain for producing PHA from CG.

## 1. Introduction

Biodiesel is a renewable fuel produced by the transesterification of vegetable oils, waste cooking oils, or animal fats [1]. It is valued for its non-toxicity, bio-based origin, biodegradability, and capacity for clean combustion [2]. These advantages have driven the rapid expansion of the biodiesel industry, which serves as a sustainable alternative to petroleum diesel, addressing challenges such as environmental pollution and resource depletion [3]. However, the growth of the biodiesel industry has led to a substantial increase in the production of crude glycerol (CG), a by-product of biodiesel production [4]. Approximately 10 kg of CG is generated for every 100 kg of biodiesel produced [5]. The composition of CG varies depending on the raw materials and catalysts used. Typically, CG comprises 60–80% glycerol along with impurities such as water, methanol, salts, free fatty acids, fatty acid methyl esters, and organic non-glycerol compounds, all of which are by-products of chemical reactions [6].

Managing CG accumulation presents significant challenges. While incineration has been proposed as a disposal method, the high boiling point of glycerol and water impurities makes it unsuitable as fuel [7]. Furthermore, purifying CG is prohibitively expensive [8]. Thermo-, physico-, and electrochemical approaches have been employed to convert CG into valuable materials, including various aldehydes, alcohols, acids, and synthetic gases [9]. Despite the inhibitory effects of CG impurities on microbial growth, biotechnological methods have emerged as the most extensively studied approach for CG valorization [10]. Biotechnological applications have successfully converted CG into high-value products such as diols, hydrogen, and β-farnesene [11,12,13].

Polyhydroxyalkanoates (PHAs) are intracellular polymers accumulated by microorganisms as carbon reserves under limited phosphorus, oxygen, or nitrogen conditions [14]. As biodegradable alternatives to conventional petrochemical-based plastics, PHAs have garnered significant interest [15]. Research has therefore focused on synthesizing PHA from CG [16]. One strategy involves engineering model strains such as *Escherichia coli* [17]. Although polyhydroxybutyrate (PHB) and poly(3-hydroxybutyrate-*co*-3-hydroxyhexanoate) (PHBHHx, p(3HB-*co*-3HHx)) have been produced from glycerol and fatty acids by introducing PHA synthesis operons into *E. coli*, the yields remain low [18]. Alternatively, screening strains that can naturally synthesize PHA from glycerol has proven more effective [19,20]. Strains such as *Cupriavidus necator* and *Burkholderia* spp. have been studied for PHA production from glycerol (Table 1). However, high-yielding strains remain rare, and most wild-type strains do not exhibit significant growth or PHB production without genetic modification.

This study aimed to isolate and characterize strains capable of high PHA production yields from CG. The Oh_219 strain may be a promising alternative for glycerol-based PHA production.

## 2. Materials and Methods

### 2.1. Screening Methods

The five sample sources included soil from the lakebed of Seoul Children’s Grand Park (37.5498170, 127.0766050) and soil from Gwaeilsan Mountain (37.5144523, 127.6087758), freshwater from Gongjicheon (37.4981866, 127.4776687), soil near the grapevine trees on Ganghwa Island (37.6261728, 126.5061005), and activated sludge from a wastewater treatment plant (36.3881622, 127.3600109). Soil samples were transferred in appropriate amounts into 50 mL conical tubes, followed by the addition of sterilized distilled water. Approximately 5 g of soil sample was added per 20 mL of distilled water. After vortexing, the samples were allowed to settle, and the supernatant was diluted 10-fold with sterile distilled water. The liquid samples (freshwater and sludge) were diluted 10-fold directly. Next, 100 µL of the diluted solution from each sample was spread onto tryptic soy agar (TSA) containing gentamycin (Gm) plates and incubated at 30 °C for 2 days. The colonies were numbered and transferred to fresh TSA-Gm plates. The numbered colonies were then pre-cultured in tryptic soy broth (TSB) containing Gm and cultured for 3 days in *Ralstonia eutropha* minimal media (ReMM) containing 1% fructose and 0.1% urea to check for PHB production. The culture broth used to confirm PHB production was analyzed using the PHA analysis method described below via gas chromatography with flame ionization detection (GC-FID). Thereafter, strains confirmed to produce PHB through GC-FID were pre-cultured from the numbered colonies. The pre-culture involved streaking the colonies onto a new TSA-Gm plate to obtain single colonies. Subsequently, single colonies were pre-cultured again in TSB-Gm, and 900 µL of the pre-culture was mixed with 600 µL of 50% glycerol and stored at −80 °C.

### 2.2. Microbial Identification and Phylogenetic Tree Construction

Identification of the strains through 16S rRNA PCR was outsourced to Bionics (Seoul, Republic of Korea). For 16S rRNA analysis, genomic DNA (gDNA) from each strain was extracted by boiling the samples with Chelex resin. Subsequently, 16S rRNA PCR was performed using bacterial gDNA and the 27F-1492R primer pair, resulting in approximately 1.5 kb PCR products. These amplified products were purified and analyzed via the Sanger method to obtain nucleotide sequences. The sequences of each strain were then analyzed using BLASTn to identify the strains.

To construct the phylogenetic tree, 16S rRNA sequences from each strain were aligned using ClustalW in the MEGA11 program. The aligned sequences were subsequently analyzed using the maximum likelihood statistical method. Phylogeny testing was conducted using the bootstrap method, employing 500 bootstrap replications. The substitution model was set to the Tamura–Nei model, and gaps/missing data were completely deleted. These settings were used to construct the phylogenetic tree.

### 2.3. Microorganisms and Culture Conditions

All PHB-producing strains, including *Burkholderia* sp. Oh_219, were pre-cultured in TSB-Gm from 20% glycerol stock stored at −80 °C. The pre-culture was streaked onto TSA-Gm plates to obtain single colonies. These streaked TSA-Gm plates were typically used for 2 weeks. The pre-culture of PHB-producing strains was grown in 5 mL TSB-Gm at 30 °C for 24 h from a single colony on TSA-Gm. For PHA production, a 5× ReMM (20 g/L NaH_2_PO_4_, 23 g/L Na_2_HPO_4_, 2.25 g/L K_2_SO_4_), 100× MgSO_4_ (39 g/L MgSO_4_), 100× CaCl_2_ (6.2 g/L CaCl_2_), and 1000× trace elements (15 g/L FeSO_4_•7H_2_O, 2.4 g/L MnSO_4_•H_2_O, 2.4 g/L ZnSO_4_•7H_2_O, and 0.48 g/L CuSO_4_•5H_2_O dissolved in 0.1 M hydrochloric acid) solution was used [35,36]. The 5× ReMM solution was sterilized by autoclaving, while the 100× MgSO_4_, 100× CaCl_2_, and 1000× solutions were filter-sterilized using a 0.22 μm polyethersulfone membrane (Sartorius, Göttingen, Germany). CG was obtained from Aekyung Chemical (Seoul, Republic of Korea). A 200 g/L glycerol or CG and 50 g/L urea solution was filtered and used as the carbon and nitrogen source, respectively. PHB production was performed at a 5 mL volume in a 14 mL round test tube. The final composition of the culture media was as follows: 4 g/L NaH_2_PO_4_, 4.6 g/L Na_2_HPO_4_, 0.45 g/L K_2_SO_4_; 0.39 g/L MgSO_4_; 0.062 g/L CaCl_2_; 0.0015 g/L FeSO_4_•7H_2_O, 0.0024 g/L MnSO_4_•H_2_O, 0.0024 g/L ZnSO_4_•7H_2_O, 0.00048 g/L CuSO_4_•5H_2_O, 20 g/L glycerol, and 1 g/L urea. In addition, various concentrations of methanol (0.25–5%), NaCl (0.25–5%), free fatty acids (0.1–1%), and PHA precursors (0.1%) were supplied, depending on the type of experiment. Unless otherwise specified, medium components were purchased from Sigma-Aldrich (St. Louis, MO, USA).

### 2.4. Analytical Methods

For PHA analysis, the culture broth was centrifuged, and the pellet was washed twice with 1 mL distilled water (DW). The cells were then transferred to a glass vial for lyophilization, and the dry cell weight was measured. Next, 1 mL of chloroform and 1 mL of 15% (*v*/*v*) H_2_SO_4_ methanol solution were added, and methanolysis was performed at 100 °C for 2 h. Afterward, the samples were cooled to room temperature. Next, 1 mL of DW was added to the methyl ester solution, and the mixture was vortexed for 5 s, twice. The bottom of the chloroform layer was transferred to a microtube containing anhydrous Na_2_SO_4_ to remove the residual water.

The filtered 1 mL sample was analyzed using a GC-FID system (Young In Chromass 6500; Young In Chromass Co., Ltd., Anyang, Republic of Korea) equipped with a fused silica capillary column (DB-FFAP, 30 mm length, 0.320 mm internal diameter, and 0.25 film; Agilent Technologies, Santa Clara, CA, USA) according to a previously described method [37,38]. The injection volume was 1 µL, and the split ratio was 1/10. Helium was used as the carrier gas at a 3.0 mL/min flow rate. The oven program for PHA analysis was as follows: 80 °C for 5 min, increased to 220 °C at a rate of 20 °C/min, and held at 220 °C for 5 min. During the analysis, the injector temperature was maintained at 210 °C, while the FID temperature was maintained at 230 °C.

### 2.5. Plasmid Construction and Transformation

*E. coli* DH5α was used as a cloning host and cultured in Luria–Bertani (LB) liquid medium or on solid medium containing 1.5% agar at 37 °C. Depending on the plasmid used, 50 µg/mL kanamycin (Km) was added. Cultures of *Burkholderia* sp. Oh_219 were grown in TSB liquid medium or on solid medium with 1.5% agar at 30 °C. For *Burkholderia* cultures, 50 µg/mL Km was added to liquid media, while 300 µg/mL Km was added to solid media, depending on the plasmid.

The pBBR1MCS2::*phaCJ* plasmid used in this study was constructed from the previously developed pBBR1MCS2::*phaC*_BP-M-CPF4_ plasmid for the synthesis of the P(3HB-*co*-3HV-*co*-4HV-*co*-5HV) tetrapolymer [39]. The *phaJ* gene derived from *Pseudomonas aeruginosa*, codon-optimized for *C. necator*, was amplified from pPhaCJ along with its ribosome binding site [40]. The amplified *phaJ* was inserted into the XbaI and SacI restriction sites of the pBBR1MCS2::*phaC*_BP-M-CPF4_ plasmid.

The constructed pBBR1MCS2::*phaCJ* plasmid was introduced into *Burkholderia* sp. Oh_219 via electroporation. A single colony of *Burkholderia* sp. Oh_219 was pre-cultured overnight in a 14 mL test tube containing 5 mL of medium, followed by an 8 h main culture in TSB. A 5 mL culture medium of the main culture was centrifuged in a 1.5 mL microtube, and the resulting cell pellet was washed twice with 20% glycerol. Subsequently, the washed pellet was resuspended in 200 μL of 20% glycerol. For electroporation, 100 μL of the cell suspension and 10 μL of the plasmid were transferred to an electroporation cuvette and subjected to EC2 mode. After electroporation, 900 μL of TSB was added, and the cells were incubated at 30 °C for 2 h for recovery. The recovery solution was centrifuged, and the resulting pellet was resuspended in 100 μL of distilled water. This suspension was plated on antibiotic-containing media.

### 2.6. Fermenter Culture Conditions

Precultures for fermentation were prepared on a 50 mL scale in two 250 mL baffled flasks at 30 °C for 24 h. Pre-culture conditions were the same as those of the main culture, and the pre-culture fluid was inoculated into the fermentor without any separate pretreatment. Fed-batch fermentation was conducted with a 2 L working volume in a 5 L fermenter. The main culture composition at the beginning of the culturing period was as follows: 4 g/L NaH_2_PO_4_, 4.6 g/L Na_2_HPO_4_, 0.45 g/L K_2_SO_4_; 0.39 g/L MgSO_4_; 0.062 g/L CaCl_2_; 0.0015 g/L FeSO_4_•7H_2_O, 0.0024 g/L MnSO_4_•H_2_O, 0.0024 g/L ZnSO_4_•7H_2_O, 0.00048 g/L CuSO_4_•5H_2_O, 20 g/L fructose, and 10 g/L urea. According to the fermentation strategy, feeding solutions containing 500 g/L glycerol and 25 g/L urea, or 250 g/L glycerol and 250 g/L urea, were supplied to maintain the glycerol concentration at 1–2%. For pH control, phosphoric acid was used as an acid and NaOH as a base. Phosphoric acid was used instead of HCl for the purpose of improving cell growth through additional P-source supply during fermentation. The pH was adjusted to 6.8, and the dissolved oxygen (DO) level was set at 40%. The initial stirring rate was 200 rpm and was increased to 600 rpm to maintain DO levels during incubation.

### 2.7. Gel Permeation Chromatography

Gel permeation chromatography (GPC) to determine the number-average molecular weight (Mn), weight-average molecular weight (MW), and polydispersity index (PDI) [41] was performed using an HPLC system (Young In Chromass Co., Ltd.) comprising a loop injector (Rheodyne 7725i; Thermo Fisher Scientific, Waltham, MA, USA), a dual-headed isocratic pump system (YL9112; Young In Chromass Co., Ltd.), a column oven (YL9131; Young In Chromass Co., Ltd.) with three columns (KG 4A, guard column; K-804 8.0 × I.D. × 300 mm; K-805, 8.0 × 300 mm; Shodex; Resonac Corporation, Tokyo, Japan), and a refractive index detector (YL9170; Young In Chromass Co., Ltd.). Chloroform was used as the mobile phase at a 1 mL/min flow rate at 40 °C. The injection volume of the prepared samples was 20 L. Polystyrene standards ranging from 5000–2,000,000 Da were used to calculate the MW and construct the calibration curve. For GPC measurement, 20 mg of PHB sample was dissolved in 2 mL chloroform at 100 °C for 1 h. Afterward, 1 mL chloroform was filtered using a 0.22 µm pore size syringe filter, and 2 µL of chloroform in which PHB was dissolved was measured using GPC.

### 2.8. Determination of Physical and Thermal Characteristics

A universal testing machine (UTM) was used to measure the tensile strength (TS), Young’s modulus (YM), and elongation at the break (EL) of the samples. For UTM measurement, the PHB film was cut into a rectangle of 6 × 1 cm and analyzed using a UTM with a gauge length of 2 cm, and the elongation at break was defined accordingly. The tests were performed at a crosshead speed of 10 mm/min. The EL was calculated using Equation (1), written as follows:

𝐸𝐿 = (𝑑_𝑎𝑓𝑡𝑒𝑟_ − 𝑑_𝑏𝑒𝑓𝑜𝑟𝑒_)/𝑑_𝑏𝑒𝑓𝑜𝑟𝑒_ × 100 (1)

where *d* represents the distance between the grips holding the sample before and after it breaks.

Differential scanning calorimetry (DSC) was used to analyze the extracted PHA films. DSC was performed using a NEXTA DSC 200 instrument (Hitachi High-Tech Corporation, Kawasaki, Japan) to analyze the thermal properties of the PHA films containing 3HB and 3HHx units [42]. Approximately 5–6 mg of the PHA film was measured in an aluminum pan for DSC analysis. The experiment was conducted under N_2_. The heating and cooling rates were all at 10 °C/min, with the following temperature program: 30 °C, 7 min → −60 °C, 10 min → 190 °C, 10 min (first heating) → −60 °C, 2 min → 30 °C, 10 min → −60 °C, 10 min → 190 °C, 10 min (second heating) → −60 °C, 0 min. The glass transition (Tg), crystallization (Tc), and melting (Tm) temperatures of the polymer were determined during the second heating cycle.

## 3. Results and Discussion

### 3.1. Screening for a Strain Producing High Amounts of PHB from Glycerol

**Figure 1:**
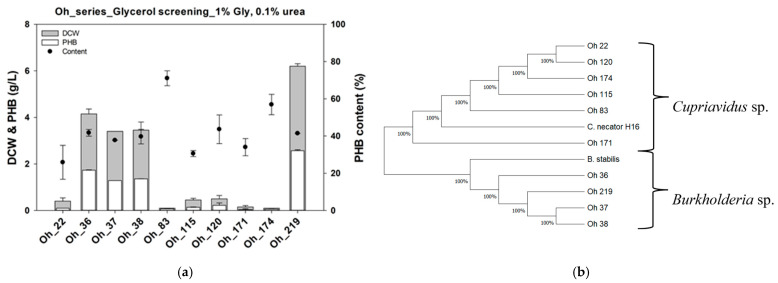
Confirmation of PHB production capacity from glycerol in 10 PHB-producing strains (**a**). Phylogenetic analysis of the 12 PHB-producing strains (**b**). PHB, polyhydroxybutyrate; DCW, dry cell weight.

*C. necator* can produce large amounts of PHB from various carbon sources, including fructose, bean oil, and CO_2_ [43]. Initially, we screened for PHB-producing strains on a minimal medium plate containing glycerol as the sole carbon source. However, no PHA-producing strains were detected. Therefore, we aimed to isolate various PHB-producing *C. necator* strains from soil, freshwater, and sludge samples in Korea using fructose and select the strain that best accumulates PHB from glycerol. Consequently, 110 strains were isolated from the 5 samples, and 10 strains that produced PHB from fructose were screened. Subsequently, the PHB production capacity from glycerol was determined for the 10 PHB-producing strains (Figure 1a). When grown in a medium containing 1% glycerol and 0.1% urea, 4 of the 10 strains produced significant amounts of PHB. Among them, the Oh_36, Oh_37, and Oh_38 strains were isolated from the moist soil collected from the mountains behind Moegaram Pension. These three strains exhibited similar PHB production, ranging from 1.28 to 1.73 g/L, with a total biomass production of 3.45–4.15 g/L. The strain with the highest biomass and PHB production was Oh_219, isolated from activated sludge. The Oh_219 strain accumulated 2.55 ± 0.05 g/L of PHB from a total biomass of 6.2 ± 0.1 g/L when grown in 1% glycerol, with a PHB content of 41%.

For strain identification, the 16S rRNA sequences of the 10 strains were analyzed, and a phylogenetic tree was constructed (Figure 1b). The 16S rRNA sequences of *C. necator* H16 (E6A55_RS08365) and *Burkholderia stabilis* (BSTAB16_RS37065) were used as reference sequences for the phylogenetic analysis. The results showed that six of the isolated strains belonged to *Cupriavidus* sp., whereas the remaining four were classified as *Burkholderia* spp. Notably, the Oh_36, Oh_37, Oh_38, and Oh_219 strains, which showed relatively high biomass and PHB production from glycerol, belonged to *Burkholderia* spp., whereas the strains that exhibited lower biomass and PHB production were classified as *Cupriavidus* sp. This suggests that the *Burkholderia* spp. isolated in Korea have higher glycerol metabolism capabilities than that of the *Cupriavidus* sp. and are, therefore, more advantageous for PHB production from glycerol. Additionally, considering the higher biomass and PHB production of the Oh_219 strain compared with those of the Oh_36, Oh_37, and Oh_38 strains, marked differences in glycerol metabolism and PHB production capabilities exist within *Burkholderia* spp. Our next objective was to characterize *Burkholderia* sp. Oh_219 and further enhance its PHB production capacity.

### 3.2. Optimization of Carbon and Nitrogen Sources

**Figure 2:**
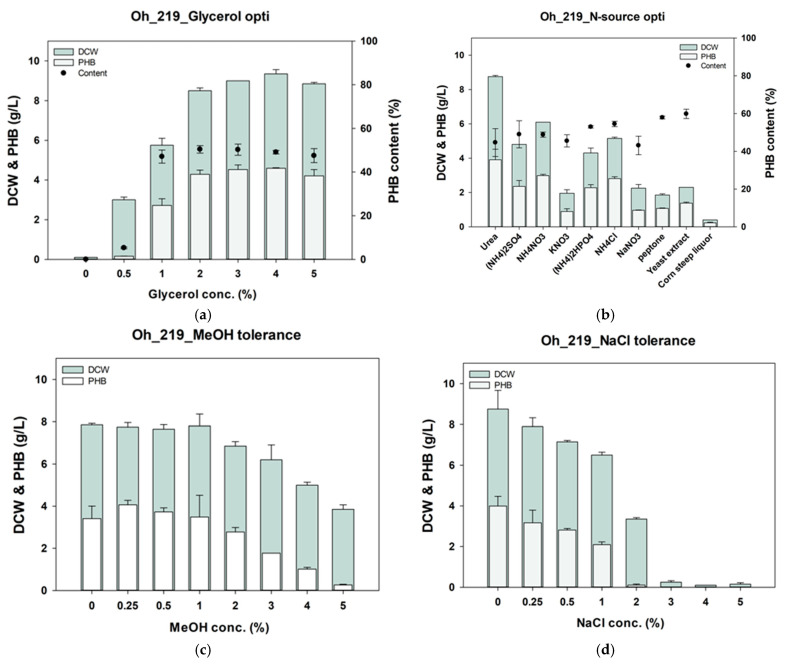
Optimization of glycerol concentration (**a**) and nitrogen source (**b**) to enhance PHB production in *Burkholderia* sp. Oh_219. Tolerance tests for methanol (**c**) and NaCl (**d**) in Oh_219 strain. PHB, polyhydroxybutyrate; DCW, dry cell weight.

PHB is a polymer that accumulates within microorganisms under carbon-rich and nitrogen-limited conditions [44]. To enhance PHB production, optimizing the carbon-to-nitrogen (C/N) ratio is crucial. Therefore, to maximize PHB production in *Burkholderia* sp. Oh_219, we optimized the C/N ratio by increasing the glycerol concentration while maintaining the urea concentration fixed at 0.1%. PHB production was confirmed at glycerol concentrations ranging from 0–5% (Figure 2a). Results showed that as the glycerol concentration increased from 0 to 2%, both dry cell weight (DCW) and PHB production increased. At 2% glycerol, the Oh_219 strain accumulated 4.25 ± 0.15 g/L of PHB from a total DCW of 8.5 ± 0.1 g/L. At glycerol concentrations above 2%, the increases in DCW and PHB production were minimal. Therefore, 2% glycerol was selected as the optimal concentration for subsequent experiments.

Nitrogen sources are crucial in the production of PHB, and the optimal nitrogen source can vary depending on the strain and the type of carbon source [45,46]. Therefore, PHB production by *Burkholderia* sp. Oh_219 was also tested in the presence of various nitrogen sources. Ten nitrogen sources, including seven synthetic (CH_4_N_2_O, (NH_4_)_2_SO_4_, NH_4_NO_3_, KNO_3_, (NH_4_)_2_HPO_4_, NH_4_Cl, and NaNO_3_) and three complex (peptone, yeast extract, and corn steep liquor) sources, were evaluated. Urea resulted in the highest production of DCW and PHB. When comparing synthetic and complex nitrogen sources, the Oh_219 strain generally showed higher DCW and PHB yields with synthetic sources despite lower yields with some of them. Although corn steep liquor, a by-product of cornstarch production, was considered more economical for PHB production, it resulted in the lowest PHB yield among the tested nitrogen sources [47].

### 3.3. Examination of the Methanol and Salt Tolerance of Oh_219

The methanol and salts present in CG are major inhibitors of microbial conversion [48]. To successfully produce PHB from CG, the strain must tolerate these inhibitors. To robustly produce PHB from CG, *Halomonas* sp. YLGW01, a salt-resistant halophile, has been considered in our previous study [49]. *Halomonas* sp. YLGW01 produced 10.5 g/L of PHB from CG in fed-batch fermentation based on its strong resistance to salts. However, since this PHB production occurred in complex media, confirming whether PHB was produced using CG as the sole carbon source is difficult. To produce PHB more economically from CG, strains that robustly produce PHB should be capable of using CG as a sole carbon source, leveraging their high resistance to methanol and salts. Therefore, to evaluate the potential of *Burkholderia* sp. Oh_219 for producing PHB from CG, its resistance to methanol and salts was evaluated using glycerol as the sole carbon source.

PHB production was evaluated in the presence of methanol at concentrations ranging from 0 to 5% (Figure 2c). The Oh_219 strain robustly produced PHB without any decrease in DCW or PHB production at methanol concentrations up to 1%. However, at 2% methanol, PHB production decreased by approximately 20% compared with that at 1% methanol. In the presence of 5% MeOH, PHB production decreased by approximately 93%. Similarly, the tolerance of Oh_219 to NaCl was tested at concentrations ranging from 0–5% (Figure 2d). As NaCl concentration increased to 1%, DCW and PHB production decreased correspondingly, with PHB production decreasing by approximately 48% at 1% NaCl. At NaCl concentrations greater than 2%, PHB production was nearly absent.

In this study, the Oh_219 strain was resistant to both methanol and NaCl up to concentrations of 1%, with PHB production decreasing with increasing concentrations. Previous reports indicate that CG typically contains 77–90% glycerol, up to 1.7% methanol, and 4.2–5.5% NaCl. Therefore, at a glycerol concentration of 2%, the initial medium may contain up to 0.04% MeOH and 0.14% NaCl. Furthermore, since the CG donated by Aekyung Chemicals used in this study contained 0.01% methanol and 0.12% salt, which are lower levels than those in typical CG, current findings suggest that these inhibitor levels will likely have minimal impact on PHB production by the Oh_219 strain.

### 3.4. Examination of Oh_219 Fatty Acid Metabolism

CG contains free fatty acids derived from various vegetable oils and their fatty acid methyl ester processing [50]. In the present study, the free fatty acid metabolism of *Burkholderia* sp. Oh_219 and the effect of fatty acids on PHB production from glycerol were evaluated.

**Figure 3:**
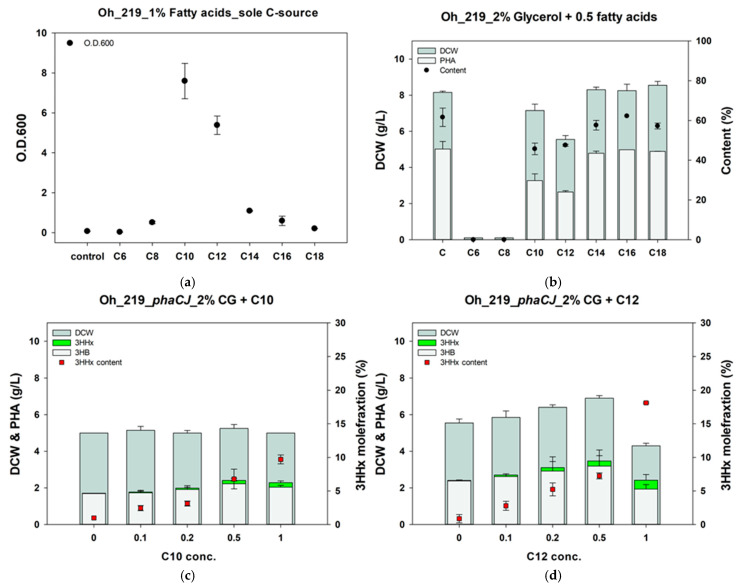
Evaluation of fatty acid metabolism in *Burkholderia* sp. Oh_219. Growth of Oh_219 with single fatty acids as the sole carbon source (**a**). PHB production by Oh_219 when single fatty acids are supplemented alongside glycerol (**b**). Assessment of P(3HB-*co*-3HHx) production in Oh_219 harboring *phaC*_BP-M-CPF4_ and *phaJ_Pa_*. P(3HB-*co*-3HHx) production by Oh_219 at various concentrations of capric (**c**) and lauric (**d**) acids added to crude glycerol. PHB, polyhydroxybutyrate; DCW, dry cell weight; C-source, carbon source; 3HB, 3-hydroxybutyrate; 3HHx, 3-hydroxyhexanoate; CG, crude glycerol.

First, we assessed the free fatty acid metabolic ability of the Oh_219 strain by measuring its OD_600_ using fatty acids with carbon chain lengths ranging from C_6_ to C_18_ as the sole carbon source (Figure 3a). A control without a carbon source was also included. The results showed that the Oh_219 strain did not grow in the presence of C_6_ but could grow with fatty acids containing eight or more carbon atoms. The growth increased with increasing carbon chain lengths from C_6_ to C_10_, with C_10_ showing the highest growth. This could be due to the lower pH of fatty acids with shorter carbon chains. However, growth decreased as the carbon chain length increased from C_12_ to C_18_, the latter being a solid fatty acid. As the melting point of fatty acids increases with longer carbon chains, they solidify more quickly upon contact with a liquid medium. Consequently, the surface areas of the fatty acids exposed to the liquid decreased as carbon chain length increased. This reduction in surface area could explain the decreased growth of the Oh_219 strain with fatty acids longer than C_12_. However, the Oh_219 strain showed higher growth with all fatty acids longer than C_12_ compared with that of the control, indicating that it can still metabolize the free fatty acids.

However, in contrast to the highest cell growth observed when C_10_ and C_12_ were used as the sole carbon sources, C_10_ and C_12_ fatty acids alongside glycerol inhibited both cell growth and PHB production (Figure 3b). However, the addition of 0.5% fatty acids with chain lengths of C_14_ or longer, together with glycerol, did not affect cell growth or PHB production. Therefore, fatty acids with chain lengths of C_14_ or longer appeared to have no negative impact on PHB production from glycerol. However, given that PHB production did not increase, it is unlikely that the Oh_219 strain metabolized these fatty acids in the presence of glycerol.

### 3.5. Production of P(3HB-Co-3HHx) by Engineered Oh_219

PHB (poly(3-hydroxybutyrate), P(3HB)) has limited industrial applications due to its brittle and rigid properties. These challenges can be addressed by incorporating other monomers into the PHA structure alongside 3HB. PHBHHx, a copolymer comprising 3HB and 3HHx monomers, has been extensively studied for its properties, which are similar to those of low-density polyethylene, a petroleum-based plastic [51]. Due to its superior properties, significant efforts have been made to synthesize PHBHHx from various carbon sources [52]. However, the primary feedstocks for PHBHHx production remain plant oils and free fatty acids [53]. This is because the introduction of *phaC*, which has high substrate specificity to 3HHx-CoA, and *phaJ*, which supplies 3HHx-CoA from the β-oxidation pathway (a fatty acid metabolic pathway), results in efficient PHBHHx synthesis [54]. Given that free fatty acids are among the main impurities in CG, PHBHHx, with its excellent properties, is expected to be produced from these free fatty acids. Although extensive research has been conducted on PHBHHx production, few studies have focused on PHBHHx production using *Burkholderia* spp. Furthermore, existing research on PHBHHx production by *Burkholderia* spp. has primarily focused on the addition of hexanoic acid [19,55,56,57,58]. To investigate whether *Burkholderia* sp. Oh_219 can produce PHBHHx from the free fatty acids in CG, the strain was genetically engineered with a vector carrying both *phaC*_BP-M-CPF4_ (known to synthesize PHBHHx) and *phaJ* from *P. aeruginosa*.

The resulting strain produced PHBHHx from CG. Oh_219 harboring *phaCJ* (Oh_219/*phaCJ*) produced PHA containing a 3HHx molar fraction of 1.0–1.3% when CG was used as the sole carbon source (Figure 3c,d). The 3HHx molar fraction in PHA was further enhanced by adding C_10_ and C_12_ fatty acids. When 2% CG was added to various concentrations of C_10_ fatty acids, the 3HHx molar fraction in the PHBHHx produced by the Oh_219/*phaCJ* strain increased to 9.7%. In the case of C_12_ fatty acids, as their concentration increased from 0 to 0.5%, the PHA yield rose from 2.4 to 3.4 g/L, and the 3HHx molar fraction increased to 7.2%. Although the PHA yield decreased at a 1% concentration of C_12_ fatty acids, the 3HHx molar fraction reached 18.1%. Taken together, the Oh_219/*phaCJ* strain was capable of producing PHBHHx from CG, with additional fatty acid supplementation effectively increasing the 3HHx molar fraction.

### 3.6. Copolymer Production Through the Addition of Various Monomers

PHB is rigid and brittle. By adding monomers other than 3-hydroxybutyrate to PHB, improving the elongation at break and lowering the melting point is possible, thereby producing a more flexible and processable polymer [59,60].

To evaluate the ability of *Burkholderia* sp. Oh_219 to synthesize various monomers, five precursors were supplied at a concentration of 0.1%: 3-hydroxypropionic acid (3-HPA), 4-hydroxybutyric acid (4-HBA), 4-hydroxyvaleric acid (4-HVA), 5-hydroxyvaleric acid (5-HVA), and lactic acid (LA) (Table 2). Oh_219 was unable to synthesize 3HP and LA from PHA. In contrast, 4HB, 3HV, 4HV, and 5HV were synthesized from PHA, but the molar fraction of 4HV was exceedingly low, while those for 4HB and 5HV were in the range of 1%. In addition to 3HB, the monomer with the highest substrate specificity was 3HV. When 4-HVA was used as a precursor, 3HV was synthesized in an 8.1% molar fraction of PHA. The synthesis of 3HV monomers from 4-HVA precursors is consistent with previous ones, wherein poly(3HB-*co*-3HV-*co*-4HV) was synthesized when 4-HVA was supplied to *C. necator* [61,62]. Furthermore, previous studies have demonstrated that the molar fractions of 3HV and 4HV in poly(3HB-*co*-3HV-*co*-4HV) vary depending on the substrate specificity of PHA synthases [39]. Therefore, the high 3HV molar fraction in Oh_219 suggests that it contains PHA synthases specific to 3HB and 3HV.

### 3.7. Comparison of PHB Production from Pure Glycerol and CG by Oh_219 in a Time-Dependent Experiment

**Figure 4:**
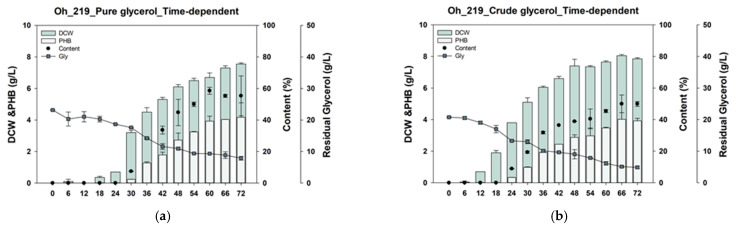
Time-dependent comparison of cell growth and PHB production by Oh_219 using pure glycerol (**a**) versus crude glycerol (**b**). PHB, polyhydroxybutyrate; DCW, dry cell weight.

The CG used in a previous study, donated by Aekyung Chemical, comprised 74.5% glycerol, 0.01% methanol, 0.12% salt, and 925.4 μg/mL protein [49]. To confirm the tolerance of the Oh_219 strain to actual CG, PHB production by the Oh_219 strain was confirmed and compared between pure glycerol (PG) and CG. When PG was used after 72 h of culture, the Oh_219 strain produced 4.15 ± 0.65 g/L of PHB in a DCW of 7.55 ± 0.05 g/L (Figure 4a). When CG was used, the Oh_219 strain produced 3.9 ± 0.1 g/L of PHB in a DCW of 7.85 ± 0.05 g/L (Figure 4b). The production of PHB by the Oh_219 strain using CG or PG did not significantly differ. Therefore, the Oh_219 strain appears to tolerate the inhibitors in CG sufficiently.

Differences in the lag phase of the Oh_219 strain between CG and PG were also compared. Oh_219 showed a shorter lag phase with CG compared with that with PG. When PG was used, Oh_219 cells grew 18 h after the start of culture. In contrast, when CG was used, Oh_219 cells began to grow 12 h after the start of culture. CG contains not only inhibitors, such as methanol and salts, but also proteins and metal ions that can promote cell growth. Therefore, the lag phase of Oh_219 using CG was shorter than that when using PG, likely due to these components in CG. Glycerol consumption by Oh_219 was higher in the presence of CG (76.5%) than of PG (66.0%). This difference in glycerol consumption may be due to the rapid growth of Oh_219 cells in CG. Taken together, the time-dependent experiment confirmed that *Burkholderia* sp. Oh_219 displayed sufficient tolerance to CG, as evidenced by its faster cell growth.

### 3.8. Fed-Batch Fermentation for the Mass Production of PHB from Glycerol

**Figure 5:**
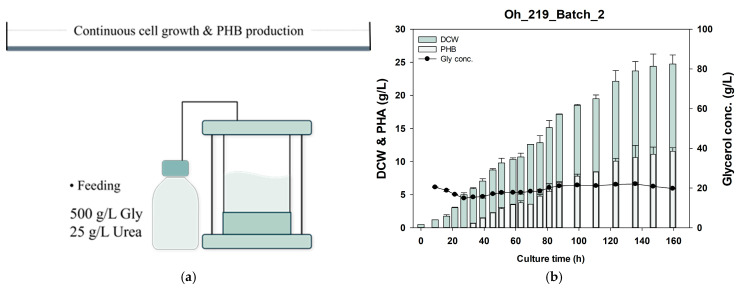

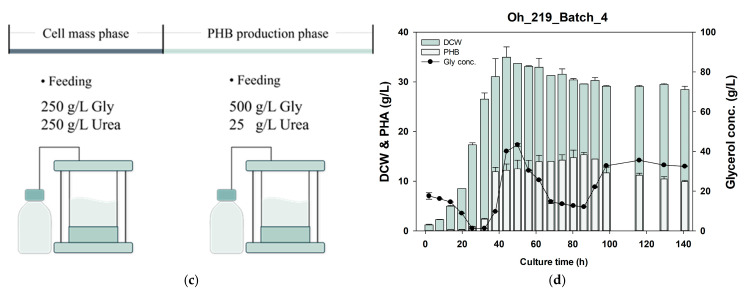
Two fermentation strategies for large-scale PHB production from glycerol. The first strategy enabled continuous cell growth and PHB accumulation by supplying glycerol and a small amount of urea (**a**,**b**). The second strategy involved a two-stage fermentation process, where glycerol and urea were fed at the same concentration to boost cell mass, followed by PHB accumulation with reduced urea supply (**c**,**d**). Gly, glycerol; PHB, polyhydroxybutyrate; DCW, dry cell weight.

Two fermentation strategies were tested for the large-scale production of PHB from glycerol using *Burkholderia* sp. Oh_219. The first strategy involved continuously supplying small amounts of nitrogen and glycerol to sustain cell growth and PHB accumulation. Typically, ammonia water is used as an alkaline pH regulator and nitrogen source; however, when urea is used as the initial nitrogen source, the pH of the medium increases, negating the need for ammonia water [21,63]. This requires switching to a nitrogen source such as (NH_4_)_2_SO_4_. Additionally, with carbon sources, such as vegetable oil (which has a high carbon content), the ammonia water supply can maintain the proper C/N ratio, allowing PHB accumulation. However, because glycerol has an exceedingly low carbon content, the supply of ammonia water may disrupt the C/N ratio and hinder PHB accumulation. Therefore, we aimed to continuously supply urea instead of ammonia to allow sustained PHB accumulation (Figure 5a). Based on previous optimization experiments, 2% glycerol and 0.1% urea were determined to be optimal; therefore, the feeding solution was prepared using 500 g/L glycerol and 25 g/L urea and supplied for 24 h. Oh_219 accumulated PHB after 24 h, and both DCW and PHB levels continued to increase until 159 h (Figure 5b). At 147 h, Oh_219 accumulated 11.05 ± 0.75 g/L of PHB in 24.4 ± 1.3 g/L of DCW, and at 159 h, it accumulated 11.55 ± 0.35 g/L of PHB in 24.75 ± 0.95 g/L of DCW.

The second fermentation strategy involved a two-stage approach that divided the process into cell mass and PHB production phases. In the cell mass phase, 250 g/L of glycerol and 250 g/L of urea significantly enhanced cell growth. After 32 h, the feed solution was switched to 500 g/L glycerol and 2.5 g/L urea to limit the nitrogen source and induce PHB accumulation (Figure 5c). At 32 h, DCW was 26.5 ± 0.9 g/L, 4.5-fold higher than that of the first fermentation strategy at the same time point (Figure 5d). Furthermore, after nitrogen limitation began, PHB accumulation commenced, peaking at 15.3 ± 0.3 g/L after 54 h of nitrogen limitation (86 h after fermentation started). Additionally, after nitrogen limitation, DCW decreased, whereas PHB accumulation steadily increased, reaching a PHA content of 53% 86 h after the start of fermentation.

In conclusion, the performance of two fed-batch fermentation strategies for increasing PHB production from glycerol using *Burkholderia* sp. Oh_219 was compared. Considering that the two-stage fermentation strategy produced 15.3 g/L of PHB in 86 h compared with 11.55 g/L of PHB in 159 h in the first strategy, the two-stage fermentation strategy offers advantages over the first strategy in terms of both production yield and time efficiency. In the present study, the PHB production yield of *Burkholderia* sp. Oh_219 was the highest among those reported in previous studies that utilized *Burkholderia* spp. to produce PHA using glycerol as the sole carbon source (Table 1).

In the case of *C. necator*, a widely studied strain, the yield of wild-type PHA was exceedingly low; however, the mutants demonstrated significantly higher PHA production during glycerol-based fermentation (Table 1). This suggests that *Burkholderia* spp. can also achieve enhanced PHA yields with further strain improvement and optimized fermentation conditions. Additionally, the PHA yield of *Burkholderia* sp. Oh_219 at the flask scale was outstanding; this strain shows great promise for PHA production from glycerol and warrants further research.

### 3.9. Comparison of the Physical Properties of PHB Produced from Fructose, PG, and CG

In the present study, *Burkholderia* sp. Oh_219 was able to produce PHB using PG and CG as well as fructose. Therefore, the equivalence of PHB produced from each carbon source was evaluated by comparing the physical properties of PHB produced when sugar and glycerol or PG and CG were used. PHB was produced from fructose, PG, and CG by *Burkholderia* sp. After extraction, the physicochemical properties of the PHB were analyzed using UTM, DSC, GPC, and a contact angle meter (Table 3).

UTM analysis revealed that all three PHB films exhibited highly rigid and brittle characteristics, as is typical for PHB. However, the film produced from CG had a notably higher tensile strength and Young’s modulus, making it more rigid. In the DSC analysis, the PHB films from fructose and PG had similar thermal properties, with Tc ranging from 108–109 °C and a Tm of 176 °C. Contrastingly, the PHB film from CG had a lower Tc of 100 °C and a Tm of 173 °C, indicating diminished thermal properties. GPC analysis showed that the PHB film from fructose had a Mw of 956,000 Da and a PDI of 1.68. However, the PG- and CG-based films had lower Mws (ca. 700,000 Da) and higher PDIs of approximately 1.9.

**Figure 6:**
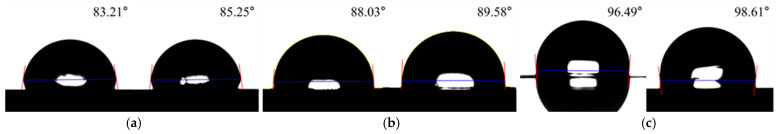
Contact angles of PHB films produced from fructose (**a**), pure glycerol (**b**), or crude glycerol (**c**) by *Burkholderia* sp. Oh_219. The blue lines represent the baseline, while the red lines indicate the tangents to the droplet’s profile.

The contact angle quantitatively represents the hydrophilicity or hydrophobicity of a polymer surface, making it a crucial factor due to its direct influence on the applicability of the polymer [64]. Since the physiochemical properties of PHB films synthesized from fructose, CG, and PG exhibited differences, we hypothesized that the hydrophobicity of these PHB films would also differ. The hydrophobicity of the films was assessed using a contact angle meter, and the results showed that the contact angle followed the order CG > PG > fructose (Figure 6). The contact angle of the CG film was the highest, averaging 97.6°, while those of the PG and fructose films were 88.8° and 84.23°, respectively. This result indicates that PHB derived from CG is more hydrophobic than those from other sources, suggesting its potential advantage for applications requiring water-resistant materials.

In conclusion, CG-derived PHB was more rigid, had a lower melting point, and exhibited a more hydrophobic surface than fructose- or PG-derived films. These differences in CG-derived PHB films could be attributed to impurities, such as fatty acids present in CG, which might leach into chloroform during extraction. Although PHB redissolution could make the CG-derived PHB film more similar to PG, the lower melting point and hydrophobic surface of the PHB film may offer advantages for PHB molding and application.

## 4. Conclusions

CG is a major by-product of the rapidly growing biodiesel industry. As biodiesel production increases, issues surrounding the accumulation and disposal of CG have escalated, leading to research aimed at converting CG into valuable materials such as PHA. However, the strains capable of efficiently producing PHB from CG are rare. In this study, 10 PHB-producing strains were isolated from soil, freshwater, and sludge samples. Among these, *Burkholderia* sp. Oh_219 was the most effective strain for producing PHB from glycerol.

Methanol and its salts are the primary microbial growth inhibitors in CG, contributing to reduced PHB yields during production. Tolerance tests demonstrated that the Oh_219 strain exhibited high methanol resistance but lower tolerance to NaCl. However, time-dependent observations of PHA production in PG and CG showed that the Oh_219 strain produced similar amounts of PHB in both substrates with a shorter lag phase in CG, indicating its tolerance to CG impurities. Oh_219 strain also capably metabolized free fatty acids, which are impurities in CG. Furthermore, genetic engineering with *phaC*_BP-M-CPF4_ and *phaJ_Pa_* enabled the Oh_219 strain to synthesize PHBHHx copolymers with a 3HHx molar fraction of 0.9% using CG fatty acids. Additional supplementation with single fatty acids significantly enhanced 3HHx content, yielding up to 9.7% 3HHx with capric acid and 18% with lauric acid. Using a two-stage fed-batch fermentation strategy in a 5 L fermenter, *Burkholderia* sp. Oh_219 produced 15.3 ± 0.3 g/L of PHB from 29.6 ± 0.1 g/L DCW in just 86 h. This is the highest reported PHA yield from glycerol using *Burkholderia* strains.

This study demonstrates the potential of *Burkholderia* sp. Oh_219 to efficiently utilize CG for the production of PHA. This provides the dual advantage of producing high-value biodegradable plastic materials while solving the CG accumulation and disposal problems. The tolerance of the Oh_219 strain to impurities in CG and its ability to metabolize fatty acids highlight its robustness under various conditions. In addition, PHBHHx produced by introducing *phaCJ* demonstrates the potential of genetically engineering this strain to produce functional polymers. Further optimization of the metabolic pathways and fermentation processes of *Burkholderia* sp. Oh_219 holds promise for significantly enhancing the efficiency of PHA production from CG, ultimately contributing to reduced production costs for these bioplastics.

Although *Burkholderia* sp. Oh_219 achieved the highest PHA yield from glycerol among the reported *Burkholderia* strains, further development is required. While Oh_219 produced PHBHHx with the introduction of external *phaCJ*, the dual expression of native and external *phaC* resulted in blended PHA with variable 3HHx molar fractions. This blended polymer has two distinct melting points, complicating its molding and processing. This issue can be addressed by developing genome editing tools for Oh_219 to delete the native *phaC*, streamline polymer composition, and improve material consistency. Future research should focus on using genetic engineering techniques to replace PHA synthases in *Burkholderia* sp. Oh_219 to achieve broader substrate specificity for copolymer synthesis. Additionally, deleting genes responsible for producing by-products, such as PHA depolymerase or exopolysaccharide-producing genes, could further increase PHA yield.

## Figures and Tables

**Table 1:** PHA production from glycerol as the sole carbon source by various reported strains.

Strain	sp.	C-Source	Culture Mode	DCW (g/L)	PHA (g/L)	Content (%)	Ref.
*Burkholderia*	*glumae* MA13	PG	Flask	4.2	1.94	46.27	[19]
		CG	Flask	3.85	1.59	41.4	
	USM (JCM15050)	PG	Flask	2.5	-	54	[20]
		CG	Flask	1.9	-	31	
	*cepacia* IPT 119	CG	Flask	1.70	1.04	-	[21]
	*cepacia* IPT 400	CG	Flask	2.04	1.17	-	
	*cepacia* BPT 1213	CG	Flask	2.41	-	66.4	[22]
		CG	Flask	5.63	3.60	64.00	
	*cepacia* ATCC 17759	CG	Flask	5.8	-	81.9	[23]
		CG	400 L fermenter	23.6	-	31	
	*sacchari* DSM 17165	PG	3.6 L fermenter	43.79	4.48	10.22	[24]
	Oh_219	PG	Flask	8.7	5.2	59.5	This study
		CG	Flask	8.1	4.0	50.0	
		PG	5 L fermenter	29.6	15.3	51.7	
*Cupriavidus*	CB15	PG	Flask	2.81	2.09	74.4	[25]
	*necator* IPT 026	CG	Flask	2.77	1.52	-	[21]
	*necator* IPT 027	CG	Flask	2.44	1.50	-	
	*necator* IPT 029	PG	Flask	2.96	1.89	63.85	[26]
		CG	Flask	2.25	1.61	71.56	
	necator ATCC 17697	CG	Flask	5.3	4.1	78	[27]
	*necator* DSM 545	CG	1 L fermenter	5.7	3.42	59.8	[28]
	*necator* DSM 545, mutant	PG	2 L fermenter	82.5	-	62	[29]
		CG	Flask	68.8	-	38	
	*necator* H16	CG	Flask	2.82	-	32.9	[30]
		PG	Flask	1.81	1.08	59.7	[31]
	*necator* H16, engineered	PG	Flask	2.25	1.44	63.9	
	*necator* H16, mutant	PG	5 L fermenter	78.9	40.7	51.6	[32]
*Rhodotorula*	*glutinis*, engineered	CG	Flask	-	0.13	4.42	[33]
		CG	Batch	-	2.87	62	
*Escherichia*	*coli*, engineered	PG	Flask	5.6	-	9.8	[34]

Abbreviation: C-source, carbon source; PG, pure glycerol; CG, crude glycerol.

**Table 2:** Identification of the PHA copolymer polymerization abilities of Oh_219 according to the precursor.

Precursor (0.1%)	DCW (g/L)	PHA (g/L)	PHA Mole Fraction (%)						
			3HB	3HP	4HB	3HV	4HV	5HV	LA
3HP	7.9	3.03	100	-	-	-	-	-	-
4HB	7.6	3.28	98.8	-	1.2	-	-	-	-
4HV	7.95	3.42	91.8	-	-	8.1	0.1	-	-
5HV	7.35	2.98	99.0	-	-	-	-	1.0	-
LA	9.05	4.83	100	-	-	-	-	-	-

Abbreviation: DCW, dry cell weight; PHA, polyhydroxyalkanoate; HV, hydroxyvalerate; HB, hydroxybutyrate; HP, hydroxypropionate; LA, lactic acid.

**Table 3:** Thermal and mechanical properties of PHB produced from fructose or glycerol by *Burkholderia* sp. Oh_219.

	UTM			DSC			GPC		
	TS (MPa)	EL (%)	YM (MPa)	Tg (°C)	Tc (°C)	Tm (°C)	Mn (10^3^)	Mw (10^3^)	PDI
Fruc	11.7 ± 1.1	11.1 ± 5.6	602 ± 33	n.a.	109	176	570	956	1.68
PG	9.9 ± 1.2	11.7 ± 2.2	413 ± 138	n.a.	108	176	404	775	1.92
CG	26.3 ± 2.1	7 ± 1.4	1225 ± 172	n.a.	100	173	382	740	1.94

Abbreviation: TS, tensile strength; EL, elongation at break; YM, young’s modulus; Fruc, fructose; PG, pure glycerol; CG, crude glycerol; UTM, universal testing machine; DSC, differential scanning calorimetry; GPC, gel permeation chromatography; Tg, glass transition temperature; Tc, crystallization temperature; Tm, melting temperature; Mn, number-average molecular weight; Mw, molecular weight; PDI, polydispersity index.

## Data Availability

All data related to the study are provided with the manuscript and its associated files.

## Abbreviations

The following abbreviations are used in this manuscript:

CG	Crude glycerol
DCW	Dry cell weight
DSC	Differential scanning calorimetry
DW	Distilled water
GPC	Gel permeation chromatography
LA	Lactic acid
MW	Molecular weight
PG	Pure glycerol
ReMM	*Ralstonia eutropha* minimal media
TSA	Tryptic soy agar
TSB	Tryptic soy broth
UTM	Universal testing machine
